# Prospective external validation of radiomics‐based predictive model of distant metastasis after dynamic tumor tracking stereotactic body radiation therapy in patients with non‐small‐cell lung cancer: A multi‐institutional analysis

**DOI:** 10.1002/acm2.14475

**Published:** 2024-08-23

**Authors:** Takanori Adachi, Mitsuhiro Nakamura, Yukinori Matsuo, Katsuyuki Karasawa, Masaki Kokubo, Takashi Sakamoto, Masahiro Hiraoka, Takashi Mizowaki

**Affiliations:** ^1^ Department of Radiation Oncology and Image‐Applied Therapy Graduate School of Medicine Kyoto University Kyoto Japan; ^2^ Department of Advanced Medical Physics Graduate School of Medicine Kyoto University Kyoto Japan; ^3^ Division of Radiation Oncology Department of Radiology Tokyo Metropolitan Cancer and Infectious Diseases Center Komagome Hospital Tokyo Japan; ^4^ Department of Radiation Oncology Kobe City Medical Center General Hospital Hyogo Japan; ^5^ Department of Radiation Oncology Kyoto Katsura Hospital Kyoto Japan; ^6^ Department of Radiation Oncology Japanese Red Cross Society Wakayama Medical Center Wakayama Japan

**Keywords:** distant metastasis, dynamic tumor tracking, lung SBRT, multi‐institutional study, prognostic prediction, prospective external validation, radiomics

## Abstract

**Background and purpose:**

This study aims to externally validate a predictive model for distant metastasis (DM) with computed tomography (CT)‐based radiomics features in prospectively enrolled non‐small‐cell lung cancer patients undergoing dynamic tumor‐tracking stereotactic body radiation therapy (DTT‐SBRT).

**Materials and methods:**

The study collected retrospective data from 567 patients across 11 institutions as the training dataset and prospectively enrolled 42 patients from four institutions as the external test dataset. Four clinical features were collected, and 944 CT‐based radiomic features were extracted from gross tumor volumes. After standardization and feature selection, DM predictive models were developed using fine and gray regression (FG) and random survival forest (RSF), incorporating clinical and radiomic features, and their combinations within the training dataset. Then, the model was applied to the test dataset, dividing patients into high‐ and low‐risk groups based on medians of risk scores. Model performance was assessed using the concordance index (*C*‐index), and the statistical significance between groups was evaluated using Gray's test.

**Results:**

In the training dataset, 122 of 567 patients (21.5%) developed DM, compared to 9 of 42 patients (21.4%) in the test dataset. In the test dataset, the C‐indices of the clinical, radiomics, and hybrid models with FG were 0.559, 0.544, and 0.560, respectively, whereas those with RSF were 0.576, 0.604, and 0.627, respectively.

The hybrid model with RSF, which exhibited the best predictive performance of all models, identified 7 of 23 patients (30.4%) as high risk and 2 of 19 patients (10.5%) as low risk for DM incidence in the test dataset (*p* = 0.116).

**Conclusion:**

Although predictive models for DM lack significance when applied to prospectively enrolled cases undergoing DTT‐lung SBRT, the model with RSF exhibits a consistent capacity to effectively classify patients at a high risk of developing DM.

## 1 INTRODUCTION

Stereotactic body radiation therapy (SBRT) is considered a recognized and effective treatment option for early‐stage non‐small‐cell lung cancer (NSCLC). Numerous clinical trials have consistently shown that precise delivery of extremely high doses, while simultaneously minimizing exposure to normal surrounding tissues, leads to high local control rates.^1^, ^2^, ^3^

The management of respiratory motion is important in lung SBRT owing to the inherent movement of lung tumors during respiration. In cases where all moving tumors are encompassed within the irradiated area, larger volumes of healthy lung tissue receive radiation doses, potentially resulting in severe adverse events like radiation pneumonitis (RP).^4^ Various techniques have been developed to address respiratory motion, including breath‐holding (BH), respiratory gating, and dynamic tumor tracking (DTT) with real‐time monitoring.^5^ The American Association of Physicists in Medicine Task Group Report No. 76 recommends employing these techniques for radiotherapy in thoracic regions characterized by substantial respiratory motion.^6^

Recently, a multi‐institutional phase II study demonstrated the efficacy and safety of DTT‐SBRT in patients with NSCLC.^7^ However, some patients still experience distant metastasis (DM) after undergoing lung SBRT. With the aim of tackling this challenge, radiomics approaches have been extensively studied for use as noninvasive methods for prognostic prediction during the treatment decision‐making process. Recent research has indicated that radiomics holds a prognostic potential in lung cancer; however, various analytical methods have been reported.^8^ Several prior studies have concentrated on using computed tomography (CT) images and radiomics features to predict DM incidence in NSCLC patients who have undergone SBRT.^9^, ^10^, ^11^ Both Huynh et al.^9^ and Li et al.^10^ reported the effectiveness of CT‐based radiomics features in predicting DM incidence following lung SBRT. Nevertheless, these studies have certain limitations that primarily stem from using small patient cohorts and single‐institution studies. To address these limitations, our retrospective multi‐institutional study, encompassing a large cohort, demonstrated that radiomics features extracted from BH‐CT images successfully predicted DM incidence following lung SBRT.^11^ Notably, no prior study has prospectively predicted DM incidence after DTT‐lung SBRT using CT‐based radiomics features.

The principal objective of this study was to prospectively validate a predictive model for DM incidence in NSCLC patients following lung SBRT. This research constitutes part of a multi‐institutional phase II study, involving the prospective enrollment of patients who underwent DTT‐lung SBRT.^7^ Our study aimed to determine whether prognostic prediction based on radiomics features remains applicable when employing techniques involving different respiratory motion management strategies.

## 2 MATERIALS AND METHODS

### 2.1 Patient selection

The study included 567 patients who underwent SBRT between January 2006 and March 2016 as the training dataset (retrospectively collected from 11 institutions), and 42 patients who underwent DTT‐lung SBRT between July 2015 and January 2018, under the multi‐institutional phase II clinical trial,^7^ as the external test dataset (prospectively enrolled from four institutions). The training dataset consisted of a subset of patients included in a previous study.^11^ Although the four institutions for the test datasets overlapped with those in the training datasets, the patient cohorts used for the test and training datasets were completely different. Patients with early‐stage NSCLC were included in both the datasets, six cases of metastatic disease were excluded from the training dataset,^11^ and five cases were excluded from the test dataset.^7^ The incidence of DM was defined as progression of the disease to other organs, considering death or censoring time points. This study was approved by the Institutional Review Boards of Kyoto University Hospital and other institutions (approval number: R2564‐1). The prospectively enrolled patients were registered with the UMIN Clinical Trials Registry (UMIN000016547).

### 2.2 CT image acquisition and treatment planning

The BH‐CT at the end of exhalation and four‐dimensional CT (4DCT) exhale phase images were acquired for training and test datasets with a slice thickness of 3 mm or less. For patients in the test dataset, spherical gold markers (Disposable Gold Marker; Olympus Medical Systems, Tokyo, Japan) were placed around the tumor under bronchoscopic guidance before acquiring the CT images to execute DTT‐lung SBRT. For both datasets, gross tumor volumes (GTVs) were manually delineated on the CT images by board‐certified radiation oncologists. The patients in the training dataset were treated with 6‐MV x‐rays and prescribed doses of 48−70 Gy in four to eight fractions and 50 Gy in four fractions in the training and test dataset, respectively. A detailed explanation of the treatment planning and dose constraints is summarized in our previous studies for the training^11^ and test datasets.^7^

### 2.3 Feature extraction

A total of four clinical features were collected, and 944 CT‐based radiomic features were extracted from inside the GTVs as voxels of interest. Clinical features included age, sex, histology, and GTV size. For the extraction of radiomics features, the acquired CT images and GTV structures were first converted into nearly raw raster data files using 3D slicer (version 4.10.2; Kitware Inc., New York, NY, USA).^12^ Next, 944 radiomics features were extracted from inside the GTVs on the CT images using PyRadiomics software (version 3.0.1; Boston, MA, USA).^13^ For the feature extraction, a resampled voxel size of 1 × 1 × 1 mm^3^ and a bin width of 25 Hounsfield units were used with a function of “forced 2D extraction.” The radiomics features consisted of 14 shape‐, 18 first‐order‐, 75 texture‐, 465 (93 × 5) Laplacian of Gaussian (LoG)‐based, and 372 (93 × 4) wavelet‐based features (Table S1). The image biomarker standardization initiative defines the extracted features, such as the first‐order, gray‐level co‐occurrence matrix (GLCM), gray‐level dependence matrix, gray‐level run‐length matrix, gray‐level size zone matrix (GLSZM), and neighboring‐gray‐tone difference matrix.^14^ An LoG filter was used to smoothen and enhance the image edges. Wavelet filters were used for four decompositions in the left–right and anterior–posterior directions: LL, LH, HL, and HH.

### 2.4 Model development

**FIGURE 1:**
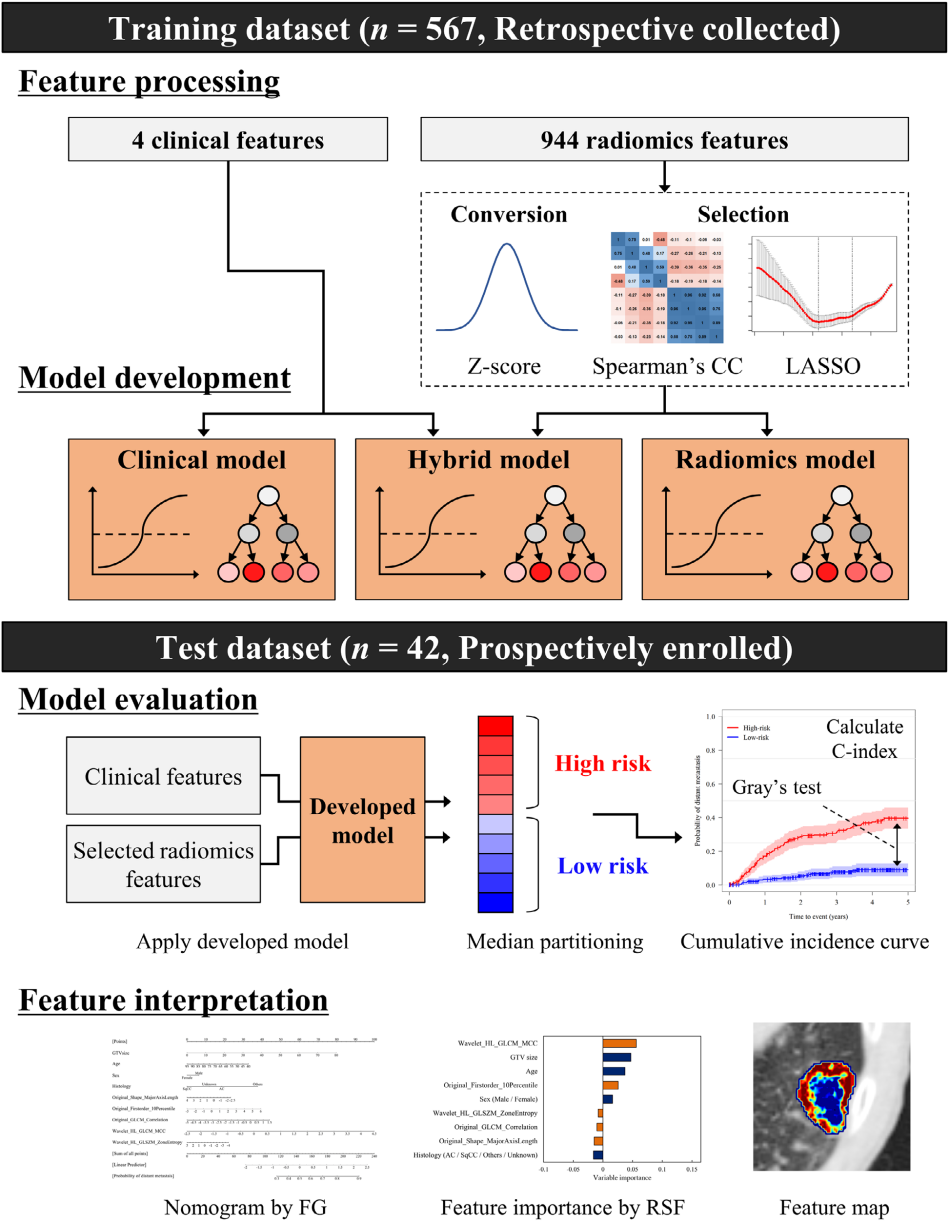
Model development overview. CC, correlation coefficient; C‐index, concordance index; FG, fine and gray regression; LASSO, least absolute shrinkage and selection operator; RSF, random survival forest.

The model development workflow is shown in Figure 1. This study was categorized as “Type 3: Development and validation using a separate data” in the “Transparent Reporting of a Multivariable Prediction Model for Individual Prognosis or Diagnosis” statement.^15^

Before model development, all radiomics features were converted into z‐scores, which were calculated with a mean of zero and a standard deviation (SD). These conversions placed the radiomics features that were expressed on a different scale on the same scale. Feature dimension reduction was achieved using Spearman's correlation coefficient (CC), followed by the least absolute shrinkage and selection operator (LASSO).^11^ Spearman's CC helps assess the redundant features, resulting in the development of a highly robust model that avoids overfitting to the training datasets. In the current study, if a radiomics feature possessed a high redundancy with another feature (CC ≥ 0.80), one of the two features with high correlation with the other remaining features was eliminated.^11^, ^16^ Then, the final set of features used for the model development was calculated by applying the LASSO to the remaining features. During the LASSO procedure, the *λ* hyperparameter was optimized to minimize the cost function of models as a feature selection role.^11^

Six models were developed using the training dataset: a clinical model with four clinical features, a radiomics model with selected radiomics features, and a hybrid model combining fine and gray regression (FG)^17^ with random survival forest (RSF).^18^ In the current study, overall survival was considered as a competing risk, and cumulative incidence curves were plotted with time‐to‐event data using FG as a statistics‐based algorithm and RSF as a machine learning‐based algorithm. The optimal hyperparameters for the RSF‐based model were determined using a stratified five‐fold cross‐validation. This process was performed to develop highly accurate predictive models and to prevent overfitting to the training datasets. The following hyperparameters were tuned: the number of variables randomly sampled at each split (*m_try_*), average number of terminal nodes (*n_ave_*), and number of trees to grow (*n_tree_*). Suitable hyperparameters were determined by searching the highest *C*‐index among the five models. Following the application of the model to the test dataset, patients were divided into high‐ and low‐risk groups based on the median values of their risk scores. These scores were computed as the sum of cumulative hazard functions from the FG‐ and RSF‐based models. When an unbalanced data proportion is considered, a majority of cases are classified in the low‐risk group, and the predictive index depends on how accurately non‐DM cases are classified in the low‐risk group. As the ultimate aim of this study was to predict the incidence of DM in patients and thus the high‐risk group, a simple median value was used to highlight these results. The corresponding description is presented in the Section 2.

### 2.5 Feature interpretation

To identify the crucial radiomics features that contributed to the model development, the nomogram and variable importance were calculated. A nomogram is a graphical tool for score calculation based on FG models. The contribution of each feature was calculated as a point score, and the probability of DM was calculated from the sum of all the points. The variable importance was calculated by noising the variables during RSF model development. Subsequently, feature color maps, which were part of a voxel‐based feature extraction function, were generated using PyRadiomics software, focusing on the predictive model with the best performance. The maps illustrate the feature visualization in the voxels of interest.

### 2.6 Statistical analysis

The model classification performance of the cumulative incidence curves was evaluated using the concordance index (*C*‐index) with 95% confidence intervals (CIs) by 2000 bootstrapping iterations. Gray's test was conducted to evaluate the statistical significance of the cumulative incidence curves between the high‐ and low‐risk groups. All statistical analyses were performed using R 4.3.0.^19^ Statistical significance was set at *p* < 0.05. The radiomics quality score provided by Lambin et al. was 32/36 (86.1%) points (Table S2).^20^

## 3 RESULTS

### 3.1 Patient characteristics

Patient characteristics are summarized in Table 1. The median follow‐up times were 2.60 (range: 0.006−11.44) and 4.44 (range: 0.24−7.09) years in the training and test datasets, respectively. A total of 122/567 (21.5%) and 9/42 (21.4%) patients developed DM in the training and test datasets, respectively. In the training and test datasets, the overall survival rates at 2 and 3 years were 78.4% and 78.6% (*p* = 0.841) and 71.2% and 73.8% (*p* = 0.856), respectively.

**TABLE 1:** Patient characteristics.

			Test dataset						
Characteristics description		Training dataset (*n* = 567)	Total (*n* = 42)	Institution A (*n* = 16)	Institution B (*n* = 5)	Institution C (*n* = 8)	Institution D (*n* = 13)	*p*‐value^a^	*p*‐value^b^
Age	Median (range) [years]	79 (41−92)	81 (49−90)	81.5 (68−90)	81 (76−85)	80 (53−88)	83 (49−90)	0.149	0.937
Sex	Male / Female	399/168	32/10	11/5	4/1	6/2	11/2	0.486	0.843
Histology	AC/SqCC/others/unknown	206/103/25/233	8/8/2/24	2/1/0/13	2/2/0/1	2/3/1/2	2/2/1/8	0.101	0.078
GTV size	Median (range) [cm^3^]	6.6 (0.2−77.7)	10.8 (1.1−65.4)	11.7 (1.8−65.4)	15.3 (4.2−43.1)	13.3 (2.9−46.7)	9.7 (1.1−45.9)	<0.05^*^	0.669
DM incidence	Number of patients (percentage)	122 (21.5%)	9 (21.4%)	1 (6.3%)	1 (20.0%)	3 (37.5%)	4 (30.8%)	1.00	0.208

*Note*: The *p*‐values corresponding to age, GTV size, sex, histology, and DM incidence were determined using independent sample *t*‐tests and Pearson contingency chi‐squared tests.

Abbreviations: AC, adenocarcinoma; DM, distant metastasis; GTV, gross tumor volume; SqCC, squamous cell carcinoma.

^a^ Comparison between training and full test datasets.

^b^ Comparison between Institutions in the Test Dataset. Bonferroni correction was applied to adjust the *p*‐values to account for multiple comparisons.

^*^ *p* < 0.05, significant difference.

### 3.2 Radiomics feature selection

From the Spearman's CC analysis, 46/944 (4.9%) radiomics features did not have high redundancy with other features (CC < 0.80). Furthermore, from the LASSO results, the remaining features were reduced to 5/944 (0.53%), (i.e., *“Original_Shape_MajorAxisLength,”* “*Original_Firstorder_10Percentile*,” “*Original_GLCM_Correlation*,” “*Wavelet_HL_GLCM_MCC*,” and “*Wavelet_HL_GLSZM_ZoneEntropy*”). The median absolute values of intraclass CCs between the GTV size and each radiomics feature were 0.04 (range: 0.01−0.08) for the training dataset and 0.02 (range: 0.01–0.06) for the test datasets. We did not conduct feature selection because there was no redundancy in the clinical features (Figure S1).

### 3.3 Performance of the predictive models

**FIGURE 2:**
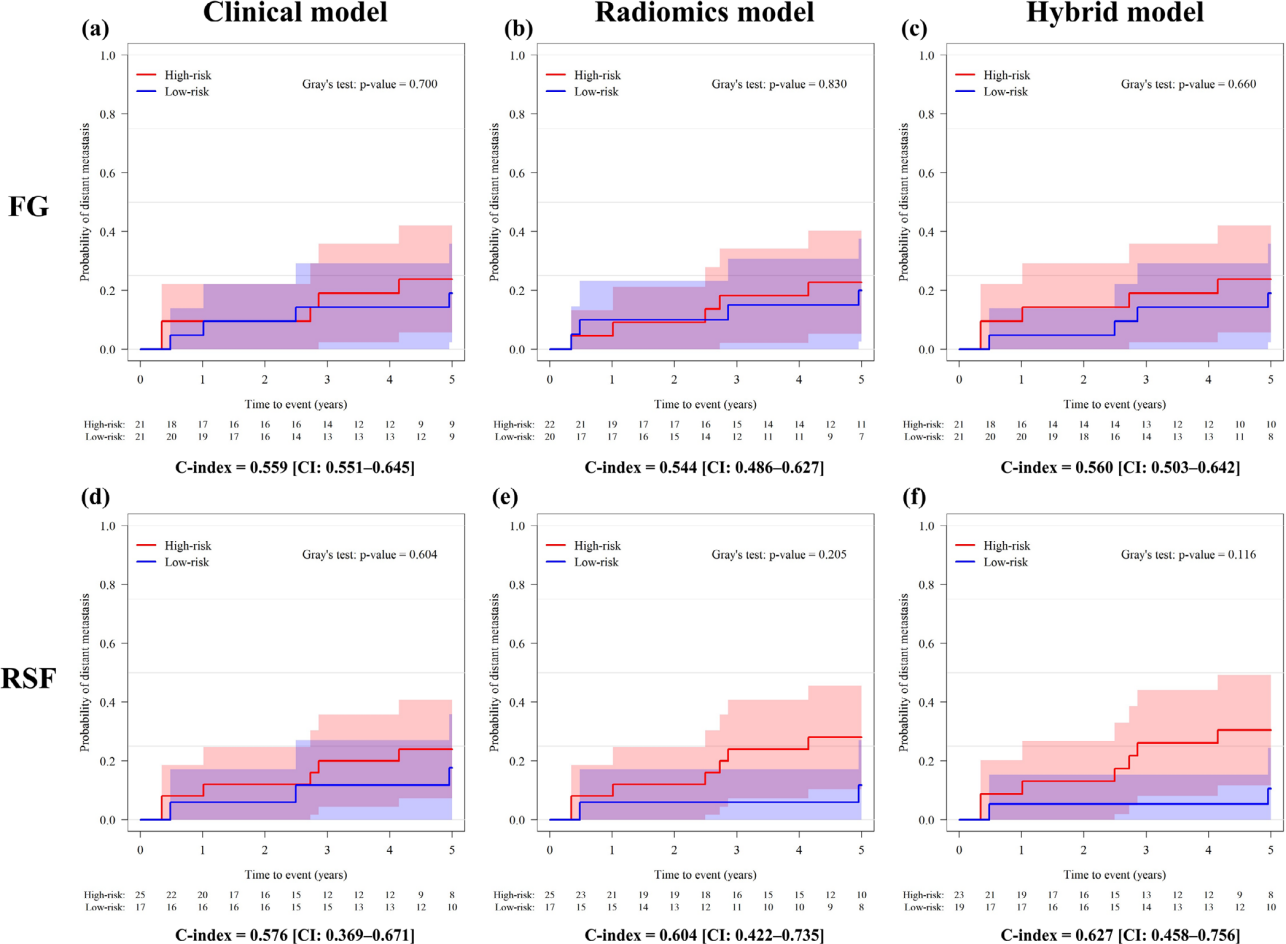
Cumulative incidence curves of (a, d) clinical, (b, e) radiomics, and (c, f) hybrid models for test datasets. (a–c) FG and (d–f) RSF. FG, fine and gray regression; RSF, random survival forest.

The cumulative incidence curves for the test datasets of the FG and RSF models are shown in Figure 2. The curves for the training dataset indicate that all FG‐ and RSF‐based models yielded a significant separation for predicting DM in the training dataset (*p* < 0.01, Figure S2). In the test dataset, the C‐indices of the clinical, radiomics, and hybrid models with FG were 0.559 [CI: 0.551−0.645], 0.544 [CI: 0.486−0.627], and 0.560 [CI: 0.503−0.642], respectively, whereas those with RSF were 0.576 [CI: 0.369−0.671], 0.604 [CI: 0.422−0.735], and 0.627 [CI: 0.458−0.756], respectively. The hybrid model with RSF exhibited the best performance among all the models. The incidence of DM in that model was 7/23 patients (30.4%) and 2/19 patients (10.5%) in the high‐ and low‐risk groups, respectively (*p* = 0.116).

### 3.4 Nomogram by FG‐based model

**FIGURE 3:**
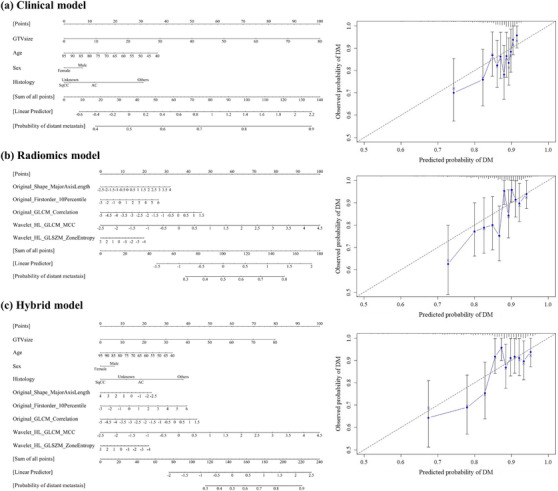
Nomogram and calibration plot. (a) Clinical model. (b) Radiomics model. (c) Hybrid model gray line in the calibration plot represents perfect prediction. AC, adenocarcinoma; GLCM, gray‐level co‐occurrence matrix; GLSZM, gray‐level size zone matrix; GTV, gross tumor volume; SqCC, squamous cell carcinoma. Note: Wavelet filters are based on four decompositions in the left–right and anterior–posterior directions: LL, LH, HL, and HH.

Figure 3 shows the results of the nomogram and calibration plot calculated using the FG‐based model. Although the calibration plot did not represent good discrimination, the most contributed features for the nomogram calculation were GTV size for the clinical model, and the *“Wavelet_HL_GLCM_MCC”* feature for the radiomics and hybrid models. Higher values of both GTV size and *“Wavelet_HL_GLCM_MCC”* corresponded with higher DM probability points.

### 3.5 Feature importance by RSF‐based model and correlated color map

**FIGURE 4:**
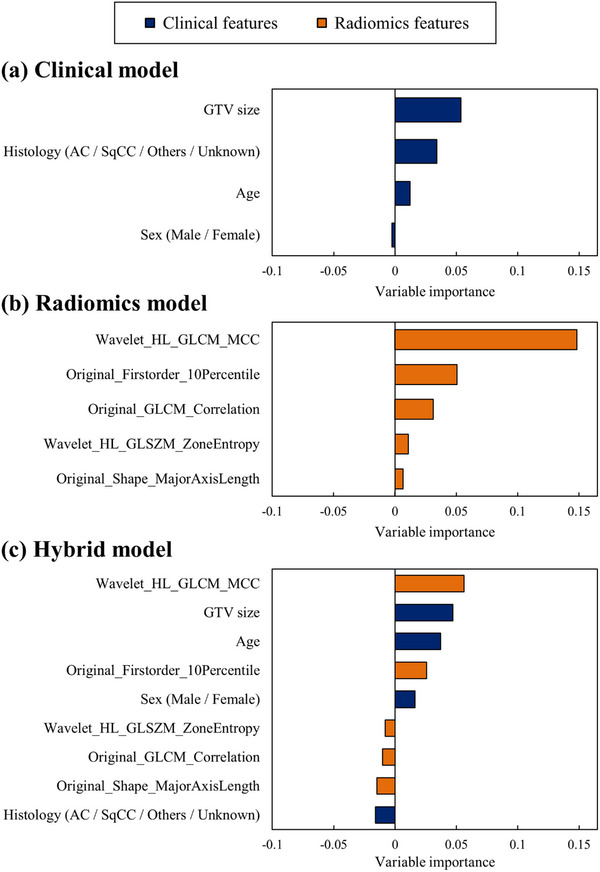
Variable importance. (a) Clinical model. (b) Radiomics model. (c) Hybrid model. Negative values indicate random calculation. AC, adenocarcinoma; GLCM, gray‐level co‐occurrence matrix; GLSZM, gray‐level size zone matrix; GTV, gross tumor volume; SqCC, squamous cell carcinoma. Note: Wavelet filters are based on four decompositions in the left–right and anterior–posterior directions: LL, LH, HL, and HH.

The results of the variable importance calculated using the RSF are shown in Figure 4. Similar to the nomogram results, the most important features were GTV size for the clinical model and the *“Wavelet_HL_GLCM_MCC”* feature for the radiomics and hybrid models. In the hybrid model, the *“Wavelet_HL_GLCM_MCC”* feature outperformed all the clinical features.

**FIGURE 5:**
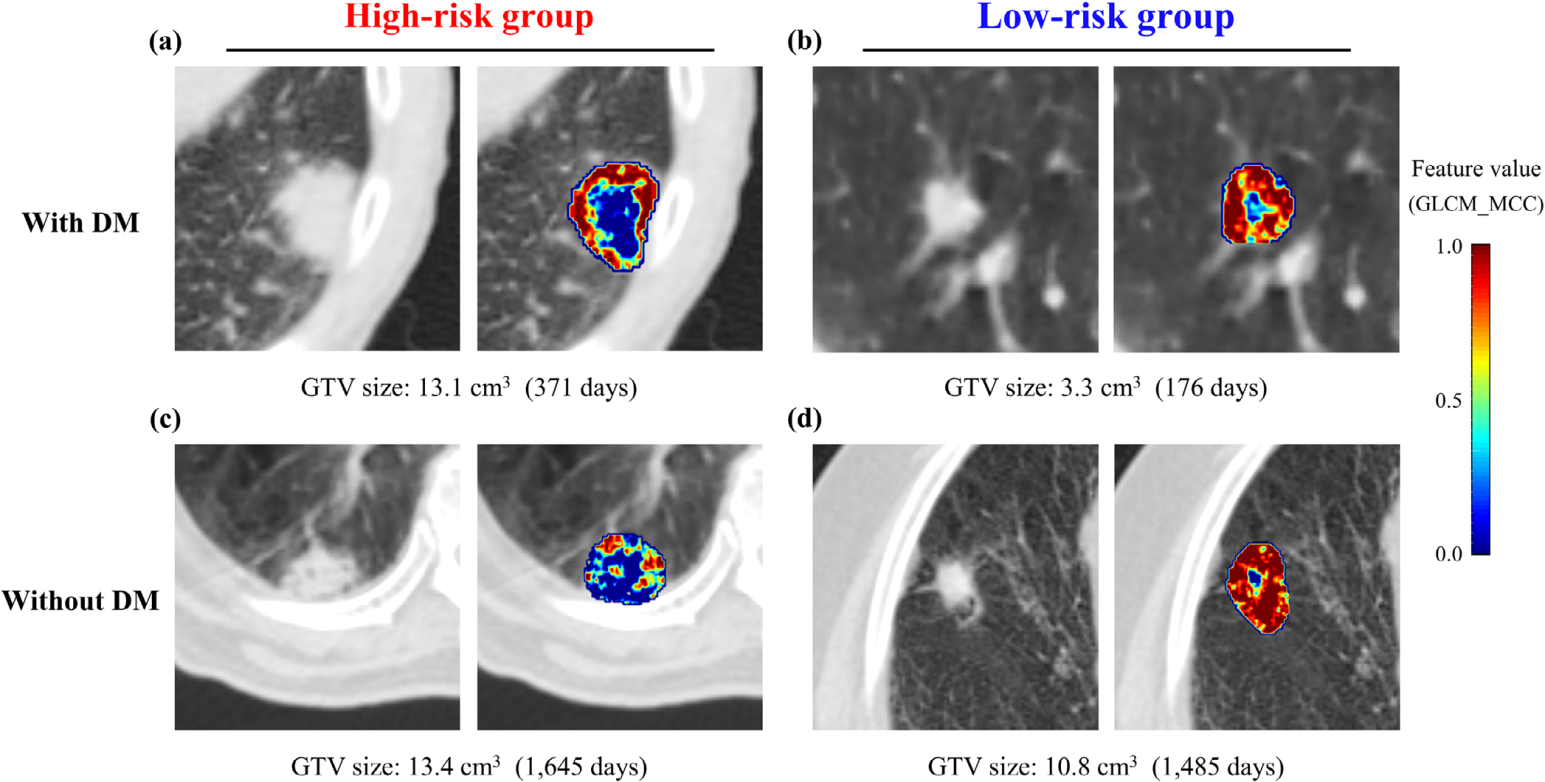
Feature color map with and without DM cases. (a) and (c): High‐risk cases. (b) and (d): Low‐risk cases patients who (a) and (b) developed DM and (c) and (d) were censored without DM. GTV size description followed by the observation date. GLCM_MCC feature values scaled to 0−1 with a color bar. DM, distant metastasis; GLCM, gray‐level co‐occurrence matrix; GTV, gross tumor volume.

Figure 5 shows an example of a feature color map calculated for the *“Wavelet_HL_GLCM_MCC”* feature for the cases with and without DM. High‐risk cases exhibited high values in the peripheral region and low values in the central region of the tumors on CT images. By contrast, low‐risk cases yielded high values for whole tumors. The median values of the above feature were higher in patients who developed DM (−0.77, range: −0.96−0.69) compared with those censored without DM (−0.35, range: −0.88−1.67) for the test dataset.

## 4 DISCUSSION

The present study externally validated a predictive model, developed from retrospectively collected cases, for its ability to predict the incidence of DM in prospectively enrolled cases. This study represents a pioneering effort because it utilized a radiomics approach to validate a DM predictive model in NSCLC patients undergoing DTT‐SBRT. Notably, both the training and test datasets were derived from a multi‐institutional cohort, with the latter consisting of prospectively enrolled DTT‐lung SBRT cases. Our findings shed new light on the application and constraints of radiomic analysis in DM prediction, particularly in the context of diverse respiratory motion management techniques employed in lung SBRT.

A previous multi‐institutional phase II study affirmed the accuracy of dose delivery in DTT‐lung SBRT for tumor control without increasing the risk of RP.^7^ A subset of DTT‐lung SBRT patients from this clinical trial was prospectively enrolled in our study. Our results revealed that despite the utilization of different irradiation techniques, the incidence of DM remained similar in retrospectively collected cases (21.5%) and prospectively enrolled cases (21.4%). Consequently, the DTT‐lung SBRT cases served as an independent external test dataset.

From the analysis of the cumulative incidence curves, it is evident that both radiomics and hybrid models outperformed clinical models when RSF was employed (Figure 2). By contrast, the predictive performance of the three models using FG was consistent. A previous study by Kakino et al. focused on retrospectively collected lung SBRT cases and reported a *C*‐index of 0.68 [CI: 0.55−0.81] for an RSF‐based hybrid model, incorporating nine clinical features and five selected radiomics features.^11^ While their study demonstrated the potential of RSF‐based models in predicting DM in NSCLC patients, it did not investigate the impact of different algorithms on predictive performance. Our results indicate that in patients with NSCLC undergoing DTT‐SBRT, all three RSF‐based models outperform the FG‐based models. Similarly, Gao et al. conducted a study using a multi‐institutional database of 1280 NSCLC patients undergoing SBRT, and they compared the discriminatory performance of FG and RSF models in predicting DM.^21^ Although their evaluation relied on the area under the curves of the receiver operating characteristic (ROC‐AUC), they found no significant difference in model performance between FG and RSF models at any time point, a result inconsistent with ours. Some possible explanations for this discrepancy may include differences in patient characteristics and the timing of DM observations. Also, ROC‐AUC may be inappropriate for evaluating time‐to‐event data because it ignores the time dimension and does not account for censoring. Therefore, it is essential to provide a detailed description of study design, duration of DM observation, and evaluation metrics when comparing the results with previous studies.

Our results identified that the most critical features were GTV size for the clinical model and “*Wavelet_HL_GLCM_MCC*” for the radiomics and hybrid models (Figures 3 and 4), in alignment with prior research.^11^ Several studies have reported a positive correlation between larger GTVs and DM development.^11^, ^22^, ^23^ Nevertheless, some patients develop DM despite possessing a smaller GTV size. In such instances, the clinical relevance of the selected radiomics feature can identify patients who are more likely to develop DM using the feature color map. The results are based on high values in the peripheral tumor and low values in the internal tumor. Although the further development of a graphical user interfere is required to integrate our findings regarding feature color maps in clinical practice, the current results show that it is possible to support the modification of treatment strategies based on the radiomics features in addition to clinical features such as GTV size.

Previous research identified “*Wavelet_LH_GLCM_MCC*” as the most crucial feature among all clinical and radiomics features.^11^ In our study, the same radiomics features were identified in the dataset used for prospective external validation, irrespective of the training dataset. These features exhibited consistent significance, even when the direction of wavelet filters with high frequency differed in the left–right and anterior–posterior directions. The GLCM_MCC feature represents complexity measured by the maximum CC.^24^ Patients with high values of these features in the peripheral tumor region were consistently classified as high risk, irrespective of irradiation technique. Therefore, visualizing feature color maps holds potential advantages and should be considered for interpreting the implications of critical radiomics features in predictive model development.

This study possesses a few limitations. First, the limited number of eligible patients in the test dataset may have reduced statistical power. However, the developed model effectively distinguishes DM cases as high‐risk groups, suggesting that this strategy may help detect DM incidence following DTT‐lung SBRT regardless of GTV size. Second, the study did not investigate the impact of imaging parameters on radiomics feature reproducibility. Previous studies have indicated that tube current has a minimal impact on feature reproducibility in CT‐based radiomics investigations.^25^ However, the differences in CT scanners have been shown to reduce reproducibility.^26^ In our study, the impact of CT scanners and imaging parameters was minimized because both training and test datasets were collected from multiple institutions. Third, the study used only manually designed radiomics features. These features have the advantage of interpretability in terms of clinical significance because they can be used in the same manner as the clinical predictors currently employed. However, the limited number of features may restrict their predictive ability. A previous study reported the effectiveness of a deep learning‐based radiomics pipeline in predicting lung nodal malignancy.^27^ Further studies are required to explore the significance of combining the above two methods. Finally, CT images for radiomics feature extraction were not standardized across training and test datasets. Although our previous phantom study suggested that target motion amplitudes less than 1.0 mm enhance reproducibility of radiomics features in thoracic regions on 4DCT‐based average intensity projection (Ave‐CT) images,^28^ the current study extracted radiomics features from BH‐CT and 4DCT phase images at the end of exhalation, potentially yielding more reproducible results than Ave‐CT images. Regardless, it is imperative to provide detailed explanations of respiratory motion management and CT images used for radiomic feature extraction in prognostic prediction studies involving thoracic regions.

## 5 CONCLUSIONS

When retrospectively derived predictive models are extrapolated to prospectively enrolled patients with early‐stage NSCLC, their ability to accurately predict DM following DTT‐lung SBRT appears to be statistically limited. Nevertheless, these models exhibit a consistent capacity to effectively classify patients at a high risk of developing DM regardless of GTV size.

## AUTHOR CONTRIBUTION

Takanori Adachi and Mitsuhiro Nakamura designed the study, the main conceptual ideas, and the proof outline. Yukinori Matsuo, Katsuyuki Karasawa, Masaki Kokubo, and Takashi Sakamoto collected the data. Takanori Adachia, Mitsuhiro Nakamura, and Yukinori Matsuo aided in interpreting the results and worked on the manuscript. Masahiro Hiraoka and Takashi Mizowaki supervised the project. Takanori Adachia wrote the manuscript with support from Mitsuhiro Nakamura. All authors discussed the results and commented on the manuscript.

## CONFLICT OF INTEREST STATEMENT

The authors have no competing financial relationships to disclose.

## Supporting information

Supporting Information

Supporting Information

Supporting Information

Supporting Information

## Data Availability

The data are not publicly available due to privacy or ethical restrictions.
