# Supplementary figures and images for: Prospective external validation of radiomics‐based predictive model of distant metastasis after dynamic tumor tracking stereotactic body radiation therapy in patients with non‐small‐cell lung cancer: A multi‐institutional analysis
Source: J Appl Clin Med Phys. 2024 Aug 23;25(10):e14475. doi: 10.1002/acm2.14475 (PMC11466494; doi:10.1002/acm2.14475)

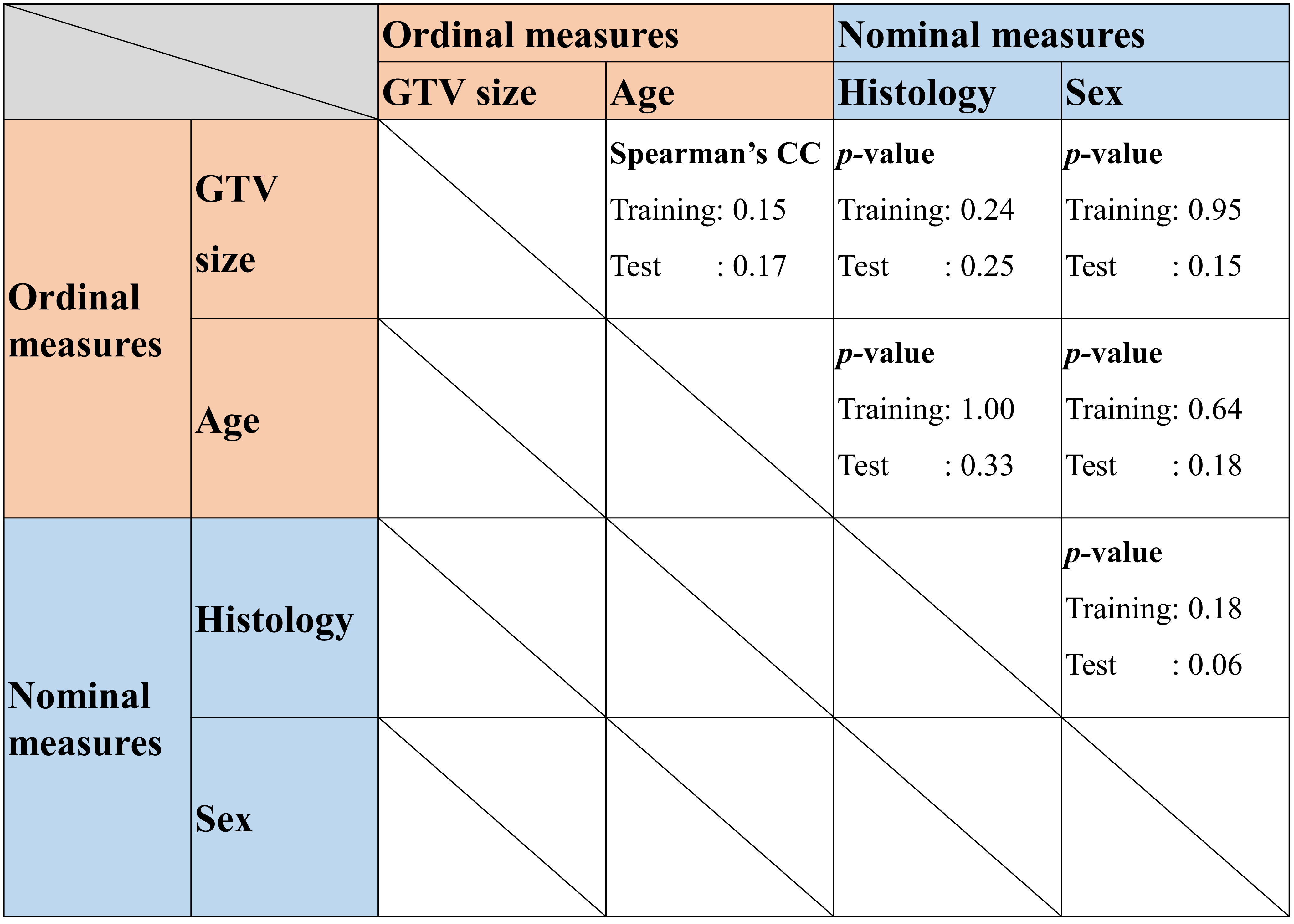

Supplement: Supplementary file 1 — Supporting Information [file ACM2-25-e14475-s003.tif]

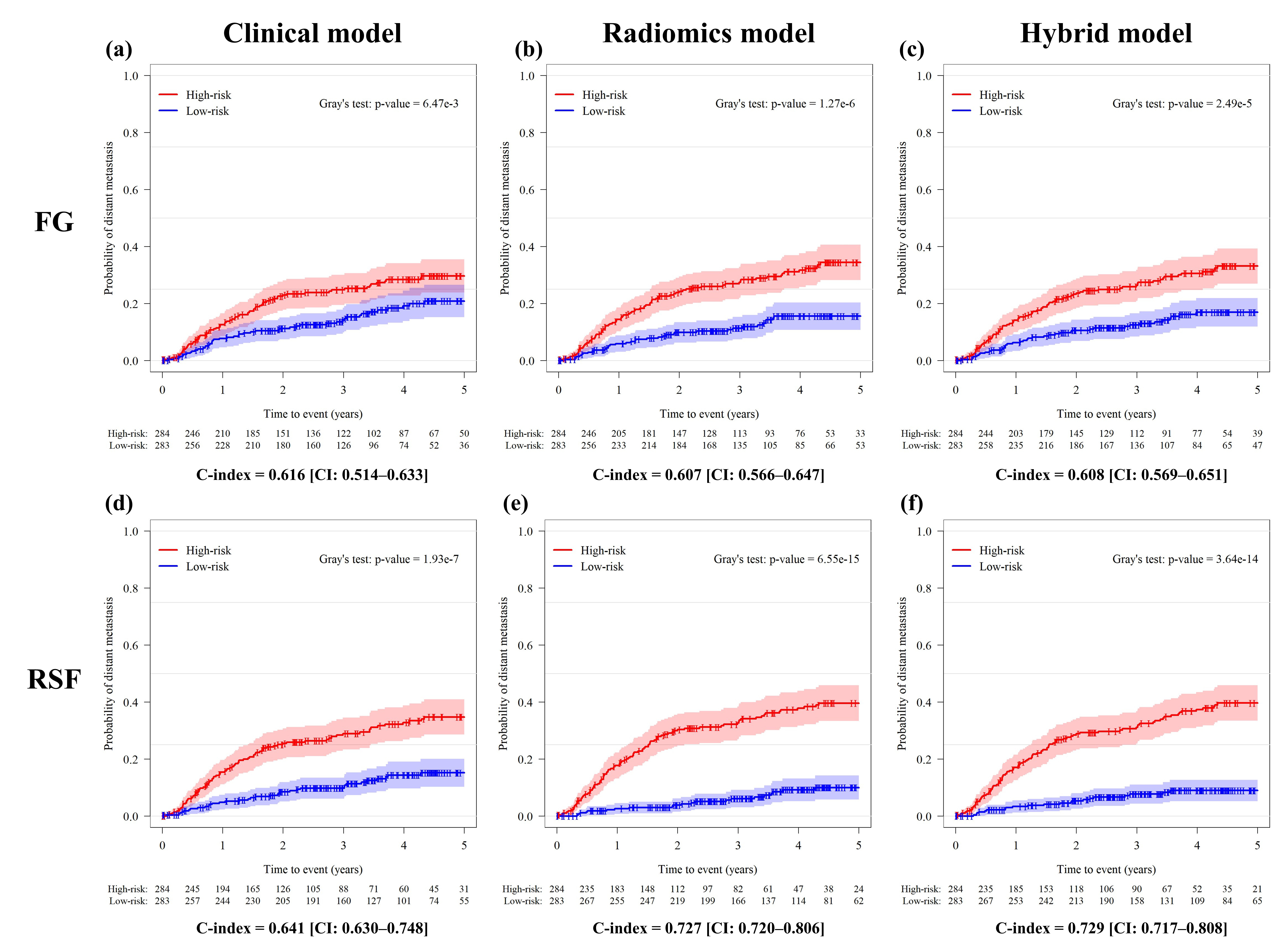

Supplement: Supplementary file 2 — Supporting Information [file ACM2-25-e14475-s004.tif]
