# Supplementary material for: Prospective external validation of radiomics‐based predictive model of distant metastasis after dynamic tumor tracking stereotactic body radiation therapy in patients with non‐small‐cell lung cancer: A multi‐institutional analysis
Source: J Appl Clin Med Phys. 2024 Aug 23;25(10):e14475. doi: 10.1002/acm2.14475 (PMC11466494; doi:10.1002/acm2.14475)
Supplement: Supplementary file 3 — Supporting Information [file ACM2-25-e14475-s002.docx]

**Supplementary Table 1: Description of radiomic features.**

| Feature types  (Number of features) | | Feature description |
| --- | --- | --- |
| Shape (14) | | Elongation, Flatness, Least Axis Length, Major Axis Length, Maximum 2D Diameter Column, Maximum 2D Diameter Row, Maximum 2D Diameter Slice, Maximum 3D Diameter, Mesh Volume, Minor Axis Length, Sphericity, Surface Area, Surface Volume Ratio, Voxel Volume |
| First order / Histogram (18) | | 10Percentile, 90Percentile, Energy, Entropy, Interquartile Range, Kurtosis, Maximum, Mean Absolute Deviation, Mean, Median, Minimum, Range, Robust Mean Absolute Deviation, Root Mean Squared, Skewness, Total Energy, Uniformity, Variance |
| Texture (75) | GLCM (24) | Autocorrelation, Cluster Prominence, Cluster Shade, Cluster Tendency, Contrast, Correlation, Difference Average, Difference Entropy, Difference Variance, Id, Idm, Idmn, Idn, Imc1, Imc2, Inverse Variance, Joint Average, Joint Energy, Joint Entropy, MCC, Maximum Probability, Sum Average, Sum Entropy, Sum Squares |
|  | GLDM (14) | Dependence Entropy, Dependence Non Uniformity, Dependence Non Uniformity Normalized, Dependence Variance, Gray Level Non Uniformity, Gray Level Variance, High Gray Level Emphasis, Large Dependence Emphasis, Large Dependence High Gray Level Emphasis, Large Dependence Low Gray Level Emphasis, Low Gray Level Emphasis, Small Dependence Emphasis, Small Dependence High Gray Level Emphasis, Small Dependence Low Gray Level Emphasis |
|  | GLRLM (16) | Gray Level Non Uniformity, Gray Level Non Uniformity Normalized, Gray Level Variance, High Gray Level Run Emphasis, Long Run Emphasis, Long Run High Gray Level Emphasis, Long Run Low Gray Level Emphasis, Low Gray Level Run Emphasis, Run Entropy, Run Length Non Uniformity, Run Length Non Uniformity Normalized, Run Percentage, Run Variance, Short Run Emphasis, Short Run High Gray Level Emphasis, Short Run Low Gray Level Emphasis |
|  | GLSZM (16) | Gray Level Non Uniformity, Gray Level Non Uniformity Normalized, Gray Level Variance, High Gray Level Zone Emphasis, Large Area Emphasis, Large Area High Gray Level Emphasis, Large Area Low Gray Level Emphasis, Low Gray Level Zone Emphasis, Size Zone Non Uniformity, Size Zone Non Uniformity Normalized, Small Area Emphasis, Small Area High Gray Level Emphasis, Small Area Low Gray Level Emphasis, Zone Entropy, Zone Percentage, Zone Variance |
|  | NGTDM (5) | Busyness, Coarseness, Complexity, Contrast, Strength |
| Laplacian of Gaussian (465) | | Sigma: 0.5, 1.0, 1.5, 2.0, 2.5 mm |
| Wavelet (372) | | Eight decompositions in the left–right, and anterior–posterior directions— LL, LH, HL, and HH |

Abbreviations: GLCM, gray-level co-occurrence matrix; GLDM, gray-level dependence matrix; GLRLM, gray-level run-length matrix; GLSZM, gray-level size zone matrix; NGTDM, neighboring gray-tone difference matrix.
