# Supplementary material for: Prospective external validation of radiomics‐based predictive model of distant metastasis after dynamic tumor tracking stereotactic body radiation therapy in patients with non‐small‐cell lung cancer: A multi‐institutional analysis
Source: J Appl Clin Med Phys. 2024 Aug 23;25(10):e14475. doi: 10.1002/acm2.14475 (PMC11466494; doi:10.1002/acm2.14475)
Supplement: Supplementary file 4 — Supporting Information [file ACM2-25-e14475-s001.docx]

**Supplementary Table 2: Calculation of radiomics quality score.**

| Criteria | Maximum [points] | Our study  [points] |
| --- | --- | --- |
| Image protocol quality | 2 | 1 |
| Multiple segmentations | 1 | 1 |
| Phantom study on all scanners | 1 | 0 |
| Imaging at multiple time points | 1 | 0 |
| Feature reduction or adjustment for multiple testing | 3 | 3 |
| Multivariable analysis with non-radiomics features | 1 | 1 |
| Detect and discuss biological correlates | 1 | 1 |
| Cut-off analyses | 1 | 1 |
| Discrimination statistics | 2 | 2 |
| Calibration statistics | 2 | 1 |
| Prospective study registered in a trial database | 7 | 7 |
| Validation | 5 | 4 |
| Comparison to 'gold standard' | 2 | 2 |
| Potential clinical utility | 2 | 2 |
| Cost-effectiveness analysis | 1 | 1 |
| Open science and data | 4 | 4 |
| Total points of radiomics quality score | 36 (100%) | 32 (86.1%) |
